# Necrotizing Glans-Sparing, Penile Corpus Spongiosum Abscess

**DOI:** 10.1055/s-0044-1801324

**Published:** 2025-01-15

**Authors:** Yog Raj Handoo

**Affiliations:** 1 Department of Plastic Surgery, RD Plastic Surgery Center, New Delhi, India

Penile abscesses are rare. 1 Most penile abscesses are in the corpus cavernosum and have a strong association with penile implants, vasoactive injection for erectile dysfunction, or addictive medicine. Fewer cases of corpus spongiosum abscesses are reported, although urinary tract infection is reported in 75 to 100% patients on long-term catheterization and has an association with urethral trauma during catheterization especially in moribund patients. We report a case of penile abscess with corpus spongiosum necrosis up to the navicular pit with sparing of the glans. Since the glans is considered a bulbar extension of the corpus spongiosum, it perishes along with the corpus spongiosum. Akyuz et al 2 presented a case report of necrosis of the corpus spongiosum and glans following catheter traction on laparoscopic radical prostatocystectomy, while Liu et al 3 reported glans necrosis on prostatic artery embolization. Christine Iilibor et al 4 reported necrotizing urethritis of infectious origin that they considered as only the second reported case.

**Fig. 1:**
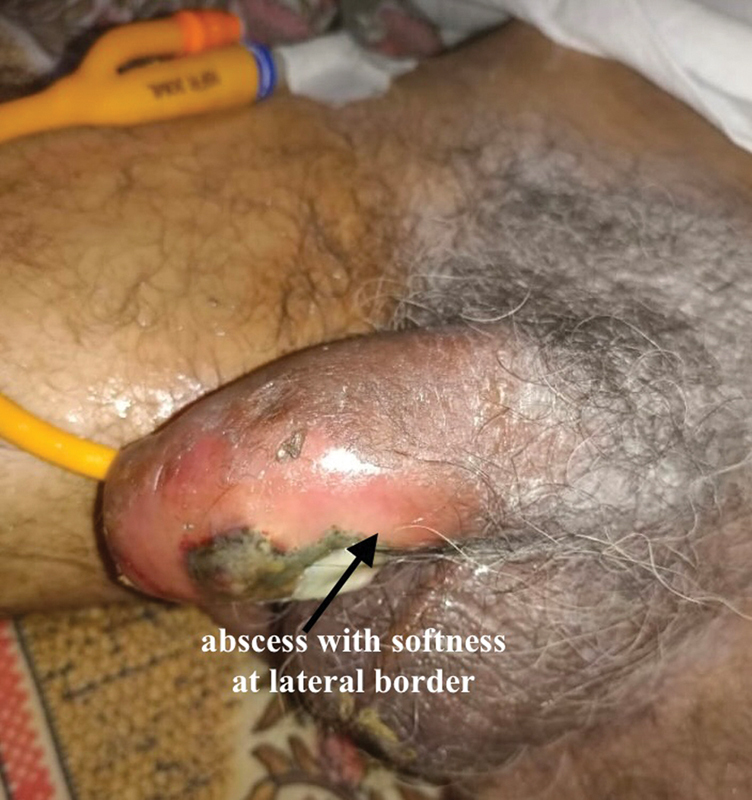
Inflamed penile area after a few days of difficult catheterization in the patient.

**Fig. 2:**
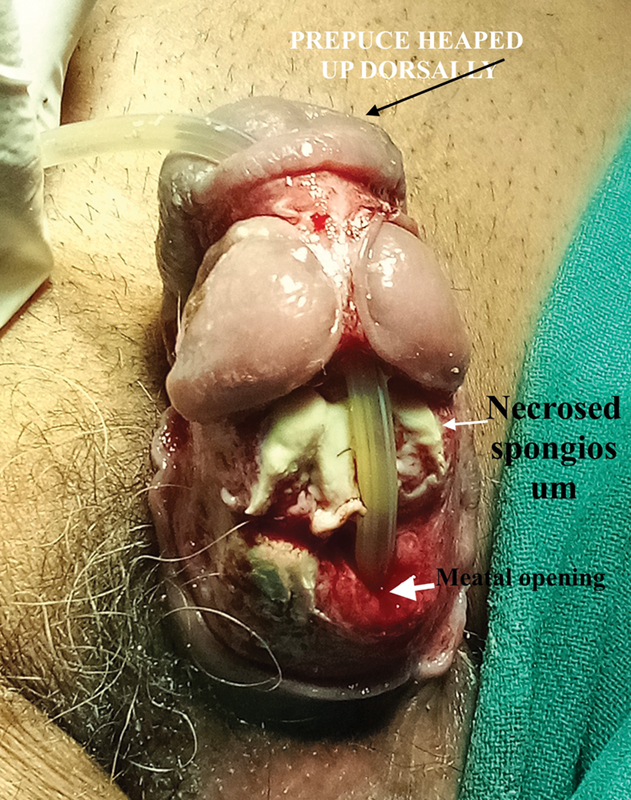
Complete necrosis of the corpora spongiosum with the catheter going behind the glans. Half of the prepuce filliped over and is held by the frenulum.

**Fig. 3:**
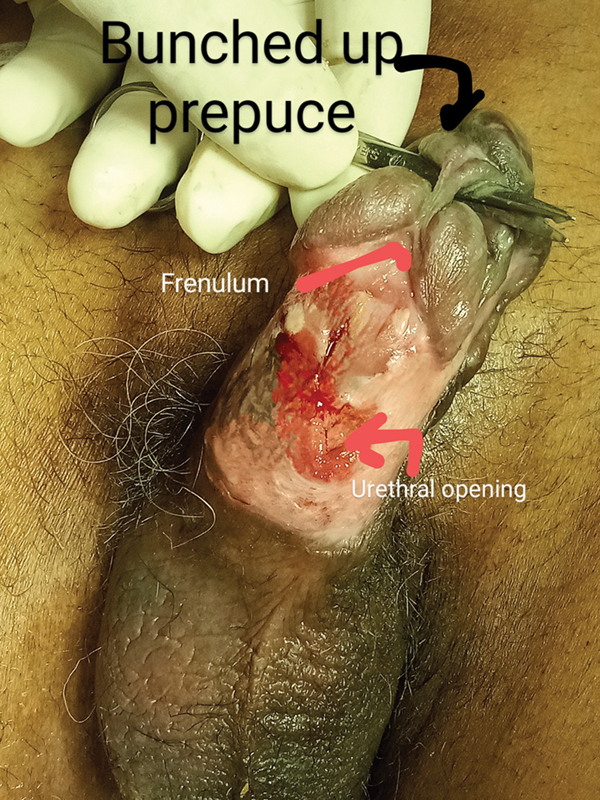
The glans separating from the corpora cavernosa, with the frenulum sawing through it.

**Fig. 4:**
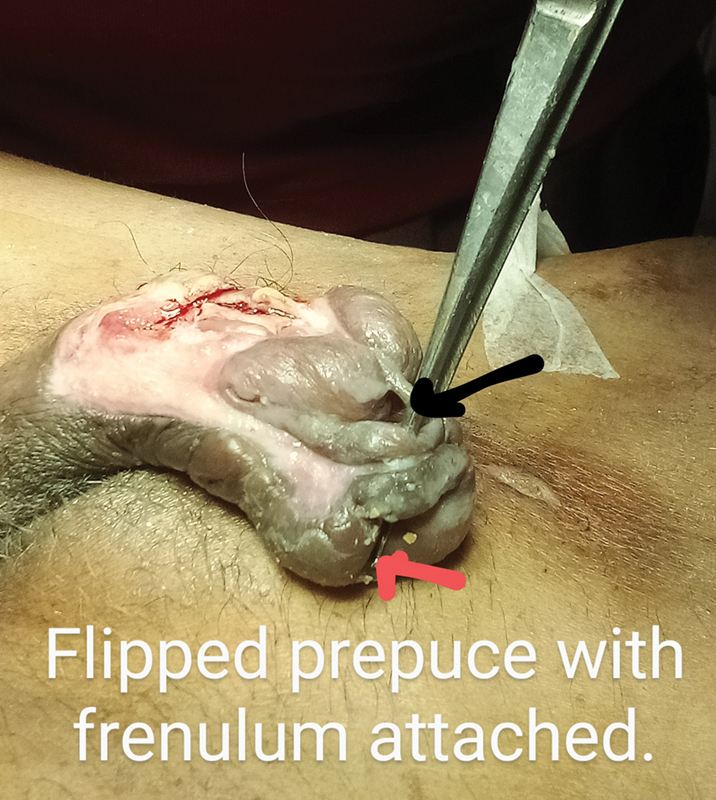
Controlled infection. Loss of corpus spongiosum from the proximal third to the external meatus. The entire prepuce flipped over and became edematous, not so pliable, and the penile skin on the dorsum of penis was also edematous and nonstretchable in the immediate aftermath of the infection.

We present the case of a 67-year-old bedridden nondiabetic man with hemiplegia on long-term catheter who had a traumatic catheter change at home. He presented with a swelling penis, with inflamed skin and 5-mm necrotic area on the left lateral aspect of the mid-penis ( Fig. 1 ). He was seen by the author at the stage when necrosis of the corpus spongiosum was already set ( Fig. 2 ) and the surgeon wanted the author to take over the case as it will need wound cover later on under penile block. 5 All ventral necrotic skin was excised along with the necrotic corpus spongiosum. Catheterization was done through the resected and debrided penile urethra ( Fig. 3 ). The glans continued to be viable throughout and started opening up the whole of the corpus spongiosum up to the necrosed navicular fossa. The distal prepuce flipped over the glans and the frenulum got stretched and flipped over the glans ( Fig. 4 ). The patient's condition improved with appropriate intravenous antibiotics and regular wound care. He was planned for perineal urethrostomy and coverage of the penile shaft with residual abundant prepuce and limited penile skin. There was fear of glans penis necrosis by damage to the dorsal penile arteries by deep degloving of the penile skin up to the perineal membrane. Skin grafting was not considered as managing the skin graft in an irritable patient would have been difficult. We feared the patient may simply avulse his dressing along with the graft. While waiting for reconstruction, the patient died of his ailments.

In summary, cases of penile corpus spongiosum necrosis 6 are rare, with only few cases reported so far. Early diagnosis and drainage are essential to prevent necrosis and morbidity.
